# 

**Published:** 1887-08

**Authors:** 


### TABLE OF CONTENTS.

ORIGINAL AND SELECTED ARTICLES.

Irrigating the Ear, by H. E Stroud, M. D, of Colorado....309
Man an Object of Zoology, by S. W. Stiles, M. D, Georgia....310
The House Fly as Distributor of Disease, by M. Moore, M. D., of Scranton, Miss....311
Gaseous Enemata, by J. Solis Cohen, M. D....312
The Effects of Transfusion upon the Heads of Decapitated Animals....315
Function of the Thyroid Gland....318
Remarkable Results of the New German Cæsarean Operations....319
Scarification in Nasal Hypertrophy, by A. G. Hobbs, M. D., Atlanta, Ga....321
Che' opodium Anthelminticum, by C. B. Maclay, M. D., Peoria, Ill....323
Poisoning by Fowler's Solution; Bismuth for Sweating Feet; Stammer.ng....324

ABSTRACTS AND GLEANINGS.

Intubation of the Larynx....325
Vesical Calculi and the Different Methods Employed for Their Removal....328
Dr. Bergeon's Method of Treating Consumption by Means of Gaseous Enemata....350
The Cure of Boils by Injection....332
Antifebrin....333
Water for Babies....334
The Cure of Epilepsy....335
Treatment of Certain Forms of Vomiting....336
Medicinal Eruptions; The Last Treatment of Phthisis....337
Treatment of Sciatica....338

PRACTICAL NOTES AND FORMULÆ.

Hypnotism; An Incandescent Gas Lamp; Exhibition of Lighting Apparats....339

A Gas Locomotive; Crystals of Gold; Magnet....340

SCIENTIFIC ITEMS.

Hematemesis; Pruritis Vulvæ; Diarrhœa of Children; For Earache....341
For Cholera Infantum; Arsenic and Lithia for Diabetes; Delirium of Typhoid Fever; Acute Tonsilitis; Acute Rheumatism....342
Intestinal Worms; Pruritus Vulvæ: Cholera Infantum; Chronic Eczema; Infantile Convulsions; Thread Worms in Children; Myrrh as a Preventive of Infectious Maladies....343

EDITORIALS AND MISCELLANEOUS.

Editorial Brevities; The International Congress....344
Stone Mountain Medical Association....345
Our Hospitals in Atlanta....347
Books and Pamphlets Received....349
Receipted; Special Notes....351

